# Income inequalities and mortality by generation among individuals with a foreign background in Sweden: a population-based study

**DOI:** 10.1016/j.lanepe.2025.101344

**Published:** 2025-06-10

**Authors:** Alexander Miething, Andrea Dunlavy, Sol P. Juárez

**Affiliations:** a Department of Public Health Sciences, Stockholm University, Stockholm, Sweden; b Centre for Health Equity Studies (CHESS), Stockholm University/Karolinska Institutet, Stockholm, Sweden

**Keywords:** Migrant, Inequality, Income inequality, Generation, Second generation, Mortality, Sweden

## Abstract

**Background:**

Evidence shows that both the mortality advantage and the lower income inequalities in mortality that characterise recent international migrants tend to disappear with time spent in the receiving country. This study examines whether absolute and relative income inequalities in mortality also increase by migrant generation in Sweden.

**Methods:**

Longitudinal data from Sweden’s population registries (2004–2018) was used to identify residents aged 25–64. An open cohort design was employed using slope (SII) and relative (RII) indices of inequality from negative binomial regressions to estimate associations between income rank position and all-cause mortality among majority population Swedes and individuals with a foreign background, classified by generation and by European or non-European origin. Sub-analyses assessed the contribution of external causes to income inequalities in mortality.

**Findings:**

Male descendants of migrants with non-European backgrounds exhibited higher relative income inequalities in mortality (ranging from RII_G2_._5_: 6.72 to RII_G2_: 11.47) than first generation non-European migrant (RII: 1.7; 95% confidence interval (CI): 1.28–2.26) and majority population men (RII: 4.73; 95% CI: 4.36–5.12). External causes accounted for 56–60% of these inequalities in mortality. Absolute income inequalities in mortality among men showed similar patterns to those observed for relative inequalities. Women showed lower absolute and relative inequalities compared to men across origins and by generation.

**Interpretation:**

Income-related inequalities in mortality appear to increase by migrant generation, particularly among men with non-European backgrounds, with external causes playing a significant role. Health and non-health targeted interventions focusing on social determinants are needed to address income inequalities in mortality.

**Funding:**

10.13039/501100006636Swedish Research Council for Health, Working Life and Welfare, and 10.13039/501100004359Swedish Research Council.

**Research in context**

**Evidence before this study**

We searched PubMed, Google Scholar, and Web of Science for studies published in English on income inequalities in mortality among international migrants and their descendants. Search terms included “∗migrant”, “inequalities”, “mortality”, “deaths”, “generation” and “second generation”, for studies published through May 2024.

Systematic reviews of the literature show that international migrants tend to have a mortality advantage relative to the receiving population. However, a growing body of evidence suggests that with increasing duration of residence, migrants tend to lose this advantage, and mortality risks may even exceed those of the receiving population. Additionally, some research indicates that the loss of migrants’ mortality advantage is associated with increasing income inequalities in mortality. Since all-cause mortality is considered to be a measure of general health at the population level, the overall evidence suggests that international migrants experience increasing health inequalities in the receiving country.

Despite a growing interest in the descendants of migrants, there is a paucity of research examining the extent to which growing income inequalities in mortality among migrants are likewise observed by migrant generation (i.e., among the descendants of migrants).

**Added value of this study**

To the best of our knowledge, this is the first study to examine absolute and relative income inequalities in mortality by migrant generation. Using nationwide registers from Sweden, we show increasing income inequalities in mortality among the descendants of international migrants and those who migrate during childhood compared to first generation adult migrants and those with Swedish ancestry. This pattern is particularly evident among men (compared to women) and among men with a non-European background. Income inequalities in mortality in these groups are mainly associated with external causes of death.

**Implications of all the available evidence**

There is evidence demonstrating that income inequalities in mortality among migrants develop, mainly among men, with increasing duration of residence in Sweden, showing a divide by European and non-European origin. Our study contributes knowledge by showing that such patterns are also evident by migrant generation. This finding is significant, as Sweden has been regarded as a favourable country for migration, with universal access to health care and welfare protections provided on equal terms to all registered residents. It therefore could be expected that income inequalities in mortality in Sweden may worsen as migration policies become more restrictive and anti-immigration sentiments increase. Yet the European/non-European divide observed in this study may also represent the development of racial and ethnic inequalities in this context. Our findings highlight the need to address migration-related inequalities in order to achieve fair and sustainable societies.

## Introduction

Social and health inequalities pose fundamental societal challenges globally.^1^ Even in egalitarian, social democratic contexts, such as the Nordic countries, persistent and growing health inequalities are well documented.^2^ In many European contexts, increasing inequalities have occurred in tandem with societies becoming more diverse, but also more restrictive towards new members. This is evidenced by increasingly stringent criteria for entry and granting permanent residence and citizenship. In receiving countries, international foreign-born migrants (hereafter migrants), often coming from lower income countries,^3^ end up occupying the most vulnerable positions in society, including the labour market, with lower incomes and fewer opportunities for social mobility for themselves and their children.^4^ Although migrants often arrive with better health than the majority population in the receiving country,^5^ evidence indicates that health tends to deteriorate with increasing duration of residence, either converging with or becoming worse than that of the majority population in the receiving context.^6^ Furthermore, recent findings suggest that health inequalities, specifically income inequalities in mortality, among migrants may actually develop in the receiving context.^7^

Studies evaluating migrant health inequalities primarily focus on first generation migrants, however inequalities associated with the migration experience have been shown to have impacts beyond an individual lifespan^8^ and to be linked with other axes of inequality in society. For example, studies focusing primarily on birth outcomes have reported growing health inequalities by generation that may be linked to the development of other forms of inequality related to race and ethnicity.^9^^,^^10^ In Sweden, an increasing number of studies have focused on the children of migrants (or the *second generation*), who have demonstrated higher mortality hazards in adulthood relative to both the first generation and the native-born without migrant parents,^11^ with external causes of death having been suggested to play a predominant role.^12^ Studies from this context have also demonstrated income inequalities among second generation groups relative to their native-born majority counterparts and to first generation migrants.^13^ However, little is known about how these two phenomena are related, that is, whether increased health risks in second generation groups may also reflect the income disadvantages they face. The role of the receiving country context in explaining health inequalities between migrants and the majority population also emerges from studies showing that migrants who arrive during childhood usually experience worse health in adulthood than those who arrive as adults and relative to the majority population.^14^ This is puzzling, as migrants who arrive during childhood should experience fewer challenges than those migrating as adults, including language barriers and essential knowledge for navigating health care systems. Due to this distinct pattern—whereby the health risks of migrant children tend to fall between those of first generation migrants and the second generation—scholars have proposed to denote this group as an intermediate migrant generation (or the 1.5 generation, G1.5).^15^

Examining and tackling health inequalities is challenging, as scholars have not reached agreement on whether we should monitor absolute or relative levels of health inequalities, and the targets set by international organisations, such as the World Health Organization, and in global initiatives, such as the Sustainable Development Goals, are not clear in this regard. Although absolute and relative measures of health inequalities are mathematically interdependent, the results from these measures often diverge,^16^ positing numerous challenges for policy makers. In this study, we examine absolute and relative income inequalities in mortality among individuals with a foreign background by migrant generation and compared to the native-born majority population (i.e., with Swedish ancestry). Additionally, we investigate the contribution of external causes of death to these inequalities. This approach provides a more comprehensive understanding of income inequalities in mortality with roots in the migration experience. Sweden’s relatively long history of immigration of individuals from several world regions provides a unique context for examining income inequalities in mortality by migrant generation.

## Methods

### Data

This study used data from various Swedish population registers, linked individually through pseudonymised personal identifiers. The Total Population Register and the Multigenerational Register were used to identify all persons registered as residents in Sweden during the follow up period from 2004 through 2018, as well as own and parents’ country of origin for those born in Sweden. An open cohort design was utilised to allow for the inclusion of individuals who turned age 25 or migrated to Sweden during the follow up period. We included all individuals born outside Sweden (G1, G1.5), who were either registered as residents at the start of the follow up (2004) or who arrived and registered in Sweden during the follow up period. For the index population, we retrieved information on cause of death from the Cause of Death Register and on income from the Longitudinal Integration Database for Health Insurance and Labour Market Studies (LISA).

The study population included individuals aged 25–64, with Swedish ancestry (native-born majority) and with a foreign background, categorised by generation: individuals born outside of Sweden who arrived as adults, aged 18 and above (G1); individuals born outside of Sweden who arrived under age 18 (G1.5); individuals born in Sweden to foreign-born parents (G2); and individuals born in Sweden to one foreign-born and one Swedish-born parent (G2.5). All groups with a foreign background were further divided by European or non-European origin, resulting in a total of nine study population subgroups. The distinction between European and non-European countries was based on geographic location only (see Appendix, p. 2 for a list of countries and regions included in each of these groups).

Censoring criteria included study entry at age 25 and exit at age 64, death, or emigration. Individuals who died in 2004 or 2005 were excluded, as these individuals did not have information on three years of income from study entry. We also excluded persons with undetermined nativity (n = 20,709) and with missing income (n = 323,074), as exclusion by income is a common method used to account for unrecorded out-migration.^17^ The final study population consisted of 6,757,547 individuals (see Appendix, p. 3 for a flowchart showing the selection and exclusion criteria that defined our study population).

### Exposure variables

The study employed relative income rank positions based on income (individualised based on total household net disposable income) as the exposure measure. This rank-based approach accommodated age- and sex-specific income distributions, and involved the computation of the Relative Index of Inequality (RII) and the Slope Index of Inequality (SII),^18^ which are widely adopted measures used for analysing socioeconomic inequalities in health. Particularly advantageous for comparing inequalities across different social strata, the RII and SII remain robust regardless of group sizes.

Income was assessed upon study entry. A three-year average of income was utilised in order to mitigate potential bias related to health selection and address potential income volatility and income reductions that may occur during periods of illness prior to death. All sources of taxable income, as well as study and social benefits, were included in the income measure.

To derive the RII, we ranked individuals by their age-, sex-, and year-specific income distribution and assigned rank values to each individual. These income rank values were then standardised by dividing them by the number of individuals in each subgroup. The resulting income rank probabilities reflect the proportional distribution of incomes in each sex- and age- and year-specific subgroup, ranging from zero for the most advantaged income group to one for the most disadvantaged income group, and were included as predictor variables in negative binomial regression models. The derived incident rate ratios (IRR) are equivalent with the RII and denote the ratio of mortality risks between the most and least advantaged income groups. An RII of one indicates no relative inequalities between the highest and lowest income groups. Values exceeding one indicate the presence of relative inequalities, whereby lower income groups have higher mortality risks. Values between zero and one indicate that higher income groups have higher mortality risks than lower income groups.

While the RII quantifies relative inequalities, the SII measures absolute differences in mortality across the entire income distribution. The SII estimates mortality differentials between the lowest and highest estimated income positions while also taking into account absolute mortality rates. Values of the SII can be positive, negative, or zero. Values of zero indicate no income inequalities in mortality rates. Positive values indicate differences in mortality rates between the most and least advantaged income groups, with the latter having the highest mortality rates. Negative values indicate differences in mortality rates between income groups whereby the most advantaged income group shows the highest mortality rates. Regardless of whether values are positive or negative, higher values indicate greater absolute inequalities in mortality rates between the most and least advantaged income groups.

The SII was derived from the RII and age-standardised mortality rates (ASMR),^7^ meaning that values should be interpreted as differences in age-standardised mortality rates. ASMRs were calculated with the direct standardisation method, using the Swedish population from 2016 as the standard population. Additional information on the calculation of the RII and SII measures is available in the Appendix (p. 4).

### Outcome and covariates

The outcome variables included all-cause mortality and mortality from external causes, assessed between January 1st 2006 and December 31st 2018. The ICD-10 codes from chapters V, W, X, and Y were used to identify external causes of death. We further distinguished external causes of mortality due to accidents (V01–X59), intentional self-harm (X60–X84), and assaults (X85–Y09). Mortality due to these external causes was used to calculate the population attributable fractions (PAF) of all deaths resulting from these causes.^19^

Age was included as a covariate and categorised into four groups: 25–34, 35–44, 45–54, and 55–64 years of age. Individuals’ sex, distinguishing between men and women, was used as a stratification variable.

### Statistical analysis

The associations between income rank probabilities and all-cause mortality, as well as the contribution of external causes of death to all-cause mortality, were assessed using negative binomial regression, as goodness-of-fit tests indicated overdispersion. In a first step, regression models in which person-years were included as the offset were used to calculate age-adjusted SIIs and RIIs in all-cause mortality for each study population subgroup, stratified by sex. Both the migrant population and their descendants are, on average, younger than the native-origin majority population. This poses a challenge when examining inequalities in mortality, as younger individuals have lower mortality risks but experience greater income inequality. To examine the impact of different age distributions across our study groups, we also conducted analyses with direct age standardisation with weights to balance the age distributions of the migrant background groups to that of the majority population.

In a second step, models (both unweighted and weighted) which excluded external causes of mortality were estimated. Based on these estimates we quantified the population attributable fractions (PAF) for continuously distributed exposures, as described in Ferguson et al.^19^ While the PAF based on the RII are expressed as percentages, inequality estimates based on the SII are presented as absolute differences in mortality rates (per 1000 person-years), since the SII reflects an absolute scale of inequality. The PAF calculations were performed separately for all external causes of death and for each specific external cause of death examined (see Appendix, p. 4 for details on the PAF calculation).

All analyses were performed in Stata V.18.0. Python V.3.11 was used to create figures. The code to reproduce the study can be found in the Appendix (pp. 15–18).

The study was approved by the Swedish Ethical Review Authority (decision no. 2021-06797-01).

### Role of the funding source

The funders of this study had no influence on the study design, data collection, data analysis, data interpretation, or writing of the manuscript.

## Results

Table 1 presents descriptive characteristics of the study population by study subgroup and sex. The highest age-standardised mortality rates (ASMRs), measured as deaths per 1000 person-years, were observed among individuals with European backgrounds (G1, G1.5, and G2). Men with non-European backgrounds (G1, G1.5, and G2.5) had slightly lower ASMRs than native-born majority and European background men. However, European G2.5 and non-European G2 and G2.5 men showed ASMRs similar to those of native-born majority men. Except for non-European G2 women, ASMRs were lower among women with non-European backgrounds compared to native-born majority women and women with European backgrounds. The lower ASMRs in some non-European background groups may partially reflect the lower mean ages of those who migrated during childhood or were born in Sweden.

**Table 1:** Descriptive characteristics of the study population, 2004–2018.

		No of individuals	Total no of deaths	No of deaths—external causes	ASMR^a^ per 1000 person-years	Age in years^b^	Annual disposable income (in 1000 SEK)^b^^,^^c^		Income rank index^b^^,^^d^
							Mean	Std dev.	
**Men**									
All		3,439,942	86,433	17,262	2.3	39.3	181.0	462.7	0.46
Native	Native-origin Swedes	2,447,307	66,230	12,831	2.3	40.4	195.1	526.3	0.51
European									
G1	European adult immigrant	239,301	5953	856	2.7	41.6	155.0	220.8	0.34
G1.5	European child immigrant	62,756	2448	511	3.3	38.6	167.3	121.4	0.44
G2	Native-born, 2 European parents	85,269	2855	810	3.0	36.9	174.6	513.1	0.47
G2.5	Native-born, 1 European parent	155,277	3607	1057	2.4	34.5	180.2	144.3	0.49
Non-European									
G1	Non-European adult immigrant	340,274	4283	635	1.7	37.1	110.0	130.4	0.22
G1.5	Non-European child immigrant	62,164	658	330	2.1	27.4	144.8	85.4	0.37
G2	Native-born, 2 Non-European parents	29,037	199	120	2.6	25.9	165.4	98.9	0.39
G2.5	Native-born, 1 Non-European parent	18,557	200	112	2.3	27.6	181.0	781.6	0.44
**Women**									
All		3,317,605	54,830	6065	1.5	39.5	142.6	265.3	0.47
Native	Native-origin Swedes	2,347,762	42,633	4429	1.5	40.6	153.3	301.8	0.51
European									
G1	European adult immigrant	254,356	4064	422	1.5	41.8	123.1	222.9	0.36
G1.5	European child immigrant	63,134	1530	203	1.9	39.7	137.4	85.2	0.47
G2	Native-born, 2 European parents	80,953	1630	283	1.8	37.1	140.0	96.6	0.50
G2.5	Native-born, 1 European parent	147,731	2134	355	1.5	34.5	145.8	117.0	0.51
Non-European									
G1	Non-European adult immigrant	322,616	2366	229	1.0	36.3	83.3	68.9	0.21
G1.5	Non-European child immigrant	56,512	315	94	1.4	27.5	122.8	72.3	0.40
G2	Native-born, 2 Non-European parents	27,207	72	25	1.6	25.9	147.1	86.5	0.44
G2.5	Native-born, 1 Non-European parent	17,334	86	25	0.9	27.6	155.1	106.0	0.50

a Age-standardised mortality rates.

b Measured at study entry.

c Three-year average of annual individualised disposable incomes (in 1000 SEK).

d Income rank probabilities ranged from ‘0’ for the most disadvantaged to ‘1’ for the most advantaged income rank.

Mean income levels varied considerably between the study subgroups. Native-born majority men had the highest income, while native-born majority women demonstrated markedly lower income. G1 represented the group with the lowest income, followed by G1.5, G2, and G2.5, across all foreign-background subgroups. Individuals with non-European backgrounds also demonstrated lower incomes compared to their European background counterparts. Sex differences in mean income were evident across all categories of migrant background, whereby women had lower incomes.

ASMRs by quartiles of income ranks are shown in the Supplementary materials (see Appendix, pp. 5–6). Among men, these findings demonstrate an inverse relationship between mortality and income ranks across all groups, with the steepest increase in mortality observed between the two lowest income quartiles (from Quartile 3 to Quartile 4), most notable in the non-European G2 group. Corresponding plots for women show fairly curvilinear increases in ASMRs across income rank quartiles.

**Fig. 1:**
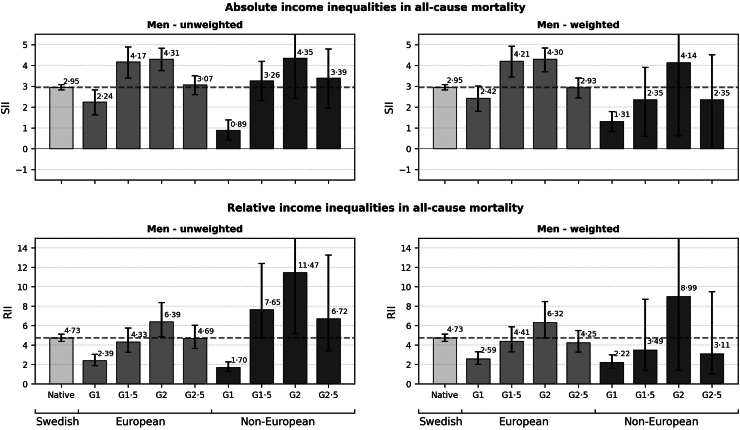
Slope indices of Inequality (SII; mortality rates per 1000 person years) and Relative Indices of Inequality (RII) in all-cause mortality by nativity background in men, aged 25–64. Dashed lines indicate estimates for native-born majority population Swedes.

**Fig. 2:**
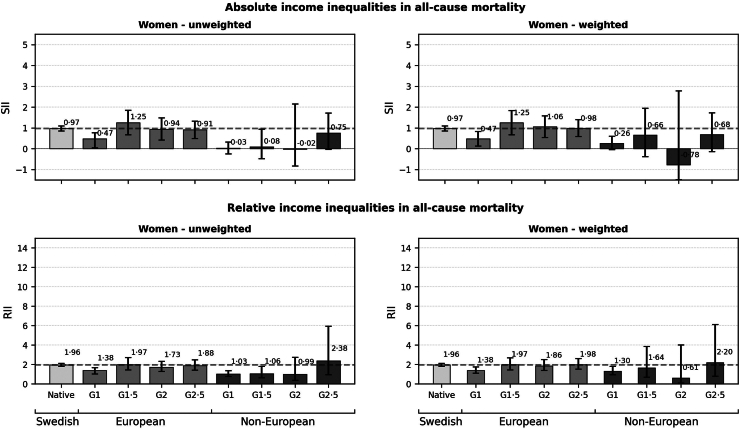
Slope indices of Inequality (SII; mortality rates per 1000 person years) and Relative Indices of Inequality (RII) in all-cause mortality by nativity background in women, aged 25–64. Dashed lines indicate estimates for native-born majority population Swedes.

Fig. 1, Fig. 2 display absolute (SII; upper panel) and relative (RII; lower panel) income inequalities in all-cause mortality among men (Fig. 1) and women (Fig. 2) across study population subgroups. Both unweighted (left panel) and weighted (right panel) results are presented in the figures (see Appendix, pp. 7–8 for estimates in tabular form). Both absolute and relative income inequalities in mortality were considerably more pronounced among men than women across all subgroups. In weighted analyses, income inequalities were of a lower magnitude in some groups compared to unweighted analyses.

Among men, the unweighted analyses revealed lower *absolute* income inequalities in mortality among G1 groups with non-European origins (SII: 0.89; 95% confidence interval (CI): 0.41–1.38) compared to native-born majority men (SII: 2.95; 95% CI: 2.82–3.08) and all other migration background subgroups. G1.5 men with European backgrounds (SII: 4.17) and G2 men with both European and non-European backgrounds (SII: 4.31, SII: 4.35, respectively) showed moderately elevated absolute income inequalities in mortality compared to the native-born majority. Fairly similar levels of absolute income inequalities were observed between native-born majority men and European G1 (SII: 2.24) and G2.5 (SII: 3.07) groups, as well as non-European G1.5 and G2.5 subgroups (SII: 3.26, SII: 3.39, respectively).

Unweighted analyses also showed patterns of findings for *relative* income inequalities among men that were largely similar to those observed for absolute inequalities, but with more pronounced differences between European and non-European background groups and relative to the native-born majority. Lower relative income inequalities in mortality were observed among G1 groups with both European and non-European origins (RII: 2.39; 95% CI: 1.88–3.04, RII: 1.7; 95% CI: 1.28–2.26, respectively) compared to native-born majority men (RII: 4.73; 95% CI: 4.36–5.12) and all other migration background subgroups. G1.5 and G2.5 men with European backgrounds showed similar magnitudes of income inequalities in mortality compared to the native-born majority, ranging from RII: 4.33 (among G1.5) to RII: 4.69 (among G2.5). G2 men with European backgrounds showed the highest magnitude of income inequalities in mortality among all groups with a European background (RII: 6.39, 95% CI: 4.87–8.38). Considerably larger income inequalities were seen among G1.5, G2, and G2.5 men with non-European origins. The highest estimates were observed among G2 (RII: 11.47; 95% CI: 5.18–25.39), followed by G1.5 (RII: 7.65; 95% CI 4.73–12.39) and G2.5 men (RII: 6.72; 95% CI: 3.4–13.26).

For both absolute and relative inequalities, the findings were largely similar in terms of direction and magnitude between unweighted and weighted analyses, yet notable differences were observed among non-European men. Although income inequalities by generation were evident in both sets of analyses, relative to native-born majority men, the weighted analyses showed lower absolute and relative income inequalities among G1.5 (SII: 2.35; RII: 3.49) and G2.5 (SII: 2.35; RII: 3.11) subgroups.

Among women, unweighted analyses showed that SIIs and RIIs were lower among European and non-European G1 groups (SII: 0.47, RII: 1.38 and SII: 0.03, RII: 1.03, respectively), compared to native-born majority women (SII: 0.97, RII: 1.96). In contrast to patterns observed for non-European men, women with non-European backgrounds showed generally weaker associations between income and all-cause mortality compared to their counterparts with European backgrounds. Weighted analyses were largely consistent with these results.

**Fig. 3:**
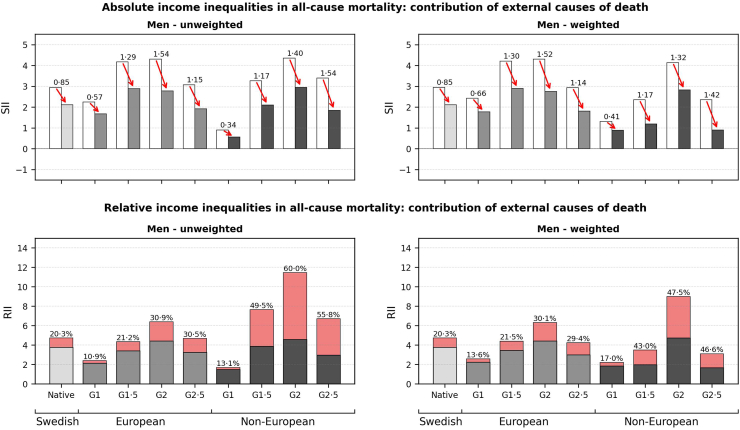
The contribution of external causes in Slope Indices of Inequality (SII) and Relative Indices of Inequality (RII) in all-cause mortality by nativity background in men, aged 25–64. The absolute portion of the SII and percentage of the RII attributable to external causes of death is displayed above the bars for each nativity subgroup.

The contribution of external causes to income inequalities in all-cause mortality among men (shown in Fig. 3) ranged from a 0.34 difference in mortality rates between the lowest and highest income rank among non-European G1 men to a 1.54 difference in mortality rates among European G2 and non-European G2.5 men. Substantial contributions of external causes were observed in relative inequalities among men with a non-European background (G1.5: 49.5%; G2: 60.0%; G2.5: 55.8%). The contribution of external causes was similar in weighted analyses. Further sub-analysis of the contributions of specific external causes of mortality (assaults, self-harm, accidents, and other external causes) showed that mortality due to accidents and self-harm made the most substantial contributions to RII estimates in unweighted and weighted analysis among men (see Appendix, pp. 9–10). Among women, negligible contributions to absolute and relative inequalities were found, with the exception of G2.5 non-European women (see Appendix, pp. 11–13).

## Discussion

This study showed that both relative and absolute income inequalities in mortality were higher for the descendants of migrants (G1.5, G2 and G2.5) compared to adult migrants (G1). However, both the magnitude and patterns of inequalities differed by sex, origin background, and generation. Specifically, our findings showed that income inequalities in mortality were lower in magnitude and more homogenous among women (across all subgroups by origin background and generation) compared to men. This result is in line with the literature which consistently shows that men are more adversely affected by income inequalities than women.^20^ Additionally, we observed large and growing inequalities across generations of men with a foreign background, but which differed considerably in magnitude by origin, with those of non-European backgrounds showing larger income-related mortality inequalities that were mainly associated with external causes of death. Taken together, our results indicate that income inequalities in mortality increase by migrant generation Sweden, with a clear divide between men with European and non-European backgrounds. This study contributes to existing knowledge by showing that increasing relative income inequalities in mortality among migrants (especially men) not only develop with longer duration of residence in Sweden^7^ but also increase by generation.

Comparisons of unweighted and age-weighted results revealed that income inequalities in mortality were impacted by differences in the age structure across study groups, mainly evident among non-European origin men. However, differences in age composition primarily influenced comparisons with the native-origin majority population. Specifically, only non-European G2 men consistently showed greater inequalities in mortality than the native-origin majority population, despite also being younger on average. This is an important finding, as studies comparing the health of migrant-origin and native-origin groups typically rely only on age adjustments, which may be insufficient to fully account for differences in health outcomes. Nonetheless, the unweighted results are relevant for informing public health interventions, which must respond to actual needs.

The fact that growing income inequalities by generation revealed a European/non-European divide is significant, because it sheds light on the possible role of inequality in linking migration background to processes of racialisation occurring in society, in line with previous studies focusing on birth outcomes in Sweden and elsewhere.^9^^,^^10^ Furthermore, our study showed that growing income inequalities in mortality by migrant generation were mainly observed among men of non-European origins, with external causes, especially accidents, playing a significant role. The contribution of external causes of death to the observed relative inequalities in this group highlights their preventable nature, rooted in social determinants. It is well-documented that migrants from middle- and low-income countries are more vulnerable to occupational fatalities, as they are overrepresented in high-risk jobs, characterised by inadequate safety measures.^21^ Our results support the importance of promoting decent and safe working conditions for all, aligning with the Agenda 2030 for Sustainable Development.

Our results also revealed variation in inequalities among second generation men (G2 and G2.5) with non-European backgrounds, depending on whether individuals have one foreign-born parent (lower inequalities) or two (higher inequalities). This finding further highlights the role of intergenerational processes, whereby ‘intermarriage’ may be seen as a marker of social integration associated with health advantages.^22^ Aligning with this body of research, our results showed that non-European G2.5 men systematically had lower mortality risks than G2 men. The differences between G2 and G2.5 may stem from the fact that persons with one native-born parent benefit from majority population social networks and social capital, potentially avoiding some forms of discrimination, such as discriminatory hiring practices tied to foreign surnames.^23^

Notably, the differences between second generation (G2 and G2.5) groups also varied by sex. Women of non-European origin showed a contrasting pattern to that observed among non-European origin men, whereby only G2.5 women exhibited higher income-related mortality inequalities compared to all other generation groups, which converged in magnitude with the inequalities observed in native-born majority population women. The magnitude of income inequality in mortality among women tends to be lower than men, given that women have lower earnings, tend to be more segregated into occupations with lower salaries, and to work part time to a greater degree than men, particularly after childbirth.^24^ Future studies should investigate the reasons why women with non-European backgrounds generally maintain their income-related mortality advantage across generations, and also explore reasons for the loss of such an advantage among the G2.5.

The mechanisms contributing to growing intergenerational income inequalities in mortality (and health in general) involve both material factors, such as lack of resources, and psychosocial factors, including perceptions of relative disadvantages and vulnerability, leading to stress and anxiety. However, determining the relative impact of both material and psychosocial factors in the observed income inequalities in mortality is outside the scope of this study. Despite this limitation, our findings clearly indicate that the roots of the inequalities observed by generation likely stem from factors unrelated to access and utilisation of healthcare services, and are instead associated with social determinants of health. Sweden’s universal healthcare system provides equal access to healthcare services for all legal residents,^25^ making it unlikely that income inequalities in mortality are driven by issues related to healthcare access. Nonetheless, migrants may still face other types of utilisation difficulties, such as language barriers, lack of knowledge of available health care services, difficulties navigating health care systems, or distrust of health care professionals.^26^ However, these challenges are expected to diminish with longer duration of residence, particularly across generations.

Although there is no consensus in the literature on whether absolute or relative measures should be used to monitor inequalities, our study demonstrated increasing inequalities by generation with both measures, with similar patterns by generation and, to a large degree, relative to the native-born. Scholars have argued that reducing relative forms of inequality represents ‘true progress’ in advancing health equity,^27^ however addressing relative inequalities is more difficult than tackling absolute inequalities, which can be more easily managed through health-related interventions. As such, our findings highlight the need for adopting a social determinants of health framework when designing strategies to address migration-related health inequalities. This approach involves considering interventions that target both health-related issues and broader social determinants, including but not limited to social and economic integration.

The use of the term ‘second generation’ is controversial and should be operationalised with caution, as *de facto* all individuals born in a given country are an inherent part of the population. The purpose of identifying the foreign-origin population by generation in this study stems from the observation that income inequalities in mortality among migrants develop with increasing duration of residence in the country,^7^ and is motivated by the aim to better understand the long-term consequences of inequalities associated with migration. Taken together, our study shows that income inequalities in mortality (and thus in health) in Sweden develop and grow by generation.

To the best of our knowledge, this is the first study to simultaneously examine income inequalities in mortality among native-born majority, migrant, and migrant descendant populations. The strengths of this study included the utilisation of longitudinal register data, which entails high coverage of the population resident in Sweden. The register information also enabled us to investigate migration background in terms of age of arrival and own or parental origin, which facilitated an intergenerational comparison of migrant background groups, including minor and adult migrants and the second generation with European and non-European origins. We were similarly able to account for the number of migrant parents among the second generation, which improved the specificity of our analysis and allowed us to speculate on possible explanatory mechanisms related to the formation of income inequalities by generation.

Yet our findings are tempered by several limitations. First, we excluded observations with missing values on income in order to reduce bias due to unrecorded emigration, which is a common limitation in migration studies. Missingness was more evident among European and Non-European migrants, and, to a lesser degree, second generation individuals with non-European backgrounds. Therefore, it is possible that the exclusion of individuals with missing income may have underestimated the income inequalities between first generation migrants compared to the native-born majority, and overestimated income inequalities by generation. However, research has suggested that complete case analysis is a valid analytical approach when missingness on the exposure variable is not random, whereas multiple imputation may result in biased estimates.^28^ Gender differences in missingness on income were negligible. Second, the migrant background groups studied are heterogenous in terms of own or parental country of origin. Despite our use of total population data, and due to our ambition to study many generational sub-groups, we were limited in the extent to which we could also account for origin or reason for migration. As a result, unaccounted variation likely exists within the broad origin categories that were employed. In fact, the smaller group sizes and number of deaths in some groups (G1.5, G2, G2.5) entailed wider confidence intervals for these mortality risk estimates, which influenced the precision of our results. Consequently, we also decided to not specifically study unaccompanied minors, as this is a very small and still quite young group. Third, although our study used register information obtained directly from administrative authorities, some degree of misclassification in terms of recorded income (e.g., unrecorded income from informal employment) or cause of death is still possible. However, such misclassification is expected to be minimal due to the high quality of the registers, and not to greatly impact our main findings. Relatedly, we decided not to control for education, which may be a relevant confounder, in our main analyses, as comparing education across generations can be challenging. Some first-generation individuals may have completed their education outside of Sweden, and for these individuals, information in the registers largely relies on self-reported or incomplete information. However, the results of an additional sensitivity analysis which accounted for education showed similar patterns of inequalities, albeit of lower magnitudes (see Appendix, p. 14). Finally, as Swedish register data does not contain information on self-reported race or ethnicity, we were not able to explore possible links between migration background and ethnicity in relation to income inequalities in mortality. This limitation also prevents us from employing intersectional perspectives which consider race and ethnicity as unique axes of inequality.

Sweden is ranked among the top ten most favourable countries for migrant integration, according to the Migrant Integration Policy Index (MIPEX),^29^ offering migrants rights equal to those granted to the native-born population. Additionally, Sweden provides universal healthcare and welfare protections for all residents.^30^ As such, our estimates of income inequalities in mortality may be conservative relative to contexts with more restrictive migration polices or less generous welfare provisions. However, Sweden’s migration policies are becoming more restrictive, similar to other European countries. As such, inequalities and associated health impacts may be more severe for upcoming generations of migrants and their children.

### Conclusion

Income inequalities in mortality increase by migrant generation, particularly among men with non-European backgrounds, for whom external causes play a significant role. Addressing income inequalities between native-born majority population Swedes and individuals with foreign backgrounds can prevent growing health inequalities that stem from migration experiences.

## Contributors

AM, AD and SPJ conceived and designed the study. AM conducted the data analysis and designed the visualisation of the results. All authors had access to and verified the data. SPJ drafted the introduction and discussion sections and AM and AD revised and made important contributions to the interpretation of the findings. All authors critically reviewed and edited the manuscript and approved the decision to submit this study for publication. AM and AD were involved in the funding acquisition.

## Data sharing statement

Data may be obtained from a third party and are not publicly available. The data used in this study were collected from Swedish administrative registers and can be requested for research use from Statistics Sweden and the Swedish National Board of Health and Welfare, provided that ethical permission has been obtained. The code to reproduce the study can be found in the Appendix (pp. 15–18).

## Declaration of interests

The authors declare that they have no conflicts of interest.
