# Supplementary material for: Income inequalities and mortality by generation among individuals with a foreign background in Sweden: a population-based study
Source: Lancet Reg Health Eur. 2025 Jun 10;55:101344. doi: 10.1016/j.lanepe.2025.101344 (PMC12179725; doi:10.1016/j.lanepe.2025.101344)
Supplement: Supplementary Methods S1 and S2, Figs. S1–S8 and Tables S1–S4 [file mmc1.docx]

**Supplementary appendix**

Supplement to: Miething A, Dunlavy A, Juárez SP. Income inequalities in mortality by generation among individuals with a foreign background in Sweden: a population-based study.

**Contents**

[**Supplementary Table S1:** List of countries and regions 2](#_Toc198642033)

[**Supplementary Figure S1:** Flowchart of the study population including selection and exclusion criteria 3](#_Toc198642034)

[**Supplementary Methods S1:** Description of RII and SII measurement construction 4](#_Toc198642035)

[**Supplementary Figure S2:** Age-standardised mortality rates per 1 000 person-years (ASMR) by income rank quartiles (men, 25-64 years). 5](#_Toc198642036)

[**Supplementary Figure S3:** Age-standardised mortality rates per 1 000 person-years (ASMR) by income rank quartiles (women, 25-64 years). 6](#_Toc198642037)

[**Supplementary Table S2:** Slope Indices of Inequality (SII; per 1 000 person-years) and Relative Indices of Inequality (RII) in mortality obtained from negative binomial regressions; men 2004 to 2018 7](#_Toc198642038)

[**Supplementary Table S3:** Slope Indices of Inequality (SII; per 1 000 person-years) and Relative Indices of Inequality (RII) in mortality obtained from negative binomial regressions; women 2004 to 2018 8](#_Toc198642039)

[**Supplementary Figure S4:** The contribution of specific external causes of death in Slope Indices of Inequality (SII), men aged 25-64. The values displayed above the bars show the absolute changes in the SII (expressed as mortality rates per 1 000 person-years) before and after accounting for the contribution of specific external causes of death within each nativity group. 9](#_Toc198642040)

[**Supplementary Figure S5:** The contribution of specific external causes of death in Relative Indices of Inequality (RII), men aged 25-64. The percentage of the RII attributable to specific external causes of death is displayed above the bars for each nativity subgroup. 10](#_Toc198642041)

[**Supplementary Figure S6:** The contribution of external causes in Slope Indices of Inequality (SII) and Relative Indices of Inequality (RII) in all-cause mortality by nativity background in women, aged 25-64. The absolute portion of the SII and percentage of the RII attributable to external causes of death is displayed above the bars for each nativity subgroup. 11](#_Toc198642042)

[**Supplementary Figure S7:** The contribution of specific external causes of death in Slope Indices of Inequality (SII), women aged 25-64. The values displayed above the bars show the absolute changes in the SII (expressed as mortality rates per 1 000 person-years) before and after accounting for the contribution of specific external causes of death within each nativity group. 12](#_Toc198642043)

[**Supplementary Figure S8:** The contribution of specific external causes of death to Relative Indices of Inequality (RII), women aged 25-64. The percentage of the RII attributable to specific external causes of death is displayed above the bars for each nativity subgroup. 13](#_Toc198642044)

[**Supplementary Table S4:** Slope Indices of Inequality (SII; per 1 000 person-years) and Relative Indices of Inequality (RII) in mortality obtained from negative binomial regressions; men and women 2004 to 2018; adjusted for education 14](#_Toc198642045)

[**Supplementary Methods S2:** Stata commands and Python code 15](#_Toc198642046)

# **Supplementary Table S1:** List of countries and regions

| **European countries and regions** | **Non-European countries and regions** |
| --- | --- |
|  |  |
| Austria | Afghanistan |
| Bosnia and Herzegovina | Africa |
| Croatia | Asia and the Pacific |
| Czechoslovakia | Brazil |
| Denmark | Chile |
| Estonia | China |
| EU28 except Nordic countries | Colombia |
| Europe except EU28 and Nordic countries | Eritrea |
| Finland | Ethiopia |
| France | India |
| Germany | Iran |
| Greece | Iraq |
| Hungary | Lebanon |
| Italy | Middle East |
| Norway | Nordic countries except Sweden |
| Poland | North America |
| Romania | Oceania |
| Russia | Pakistan |
| Soviet Union | Peru |
| Spain | Philippines |
| Sweden | Somalia |
| The Netherlands | South America |
| Yugoslavia | South Korea |
|  | Sri Lanka |
|  | Syria |
|  | Thailand |
|  | Turkey |
|  | United Kingdom and Northern Ireland |
|  | Unknown |
|  | USA |
|  | Vietnam |
|  | Republic of Vietnam |

**Total population**

Individuals born between 1940 and 1993 who were alive and recorded in the register at any point during the period 2004 to 2018.

**N = 7 114 008**

**Excluded due to missing:**

Missing income information: 323 074

Undetermined nativity background: 20 709

**Excluded by design:**

Individuals who died in 2004 and 2005: 12 678

**Study population**

**N = 6 757 547**

Total number of deaths: 141 263

Total person-years at risk: 71 472 809

# **Supplementary Figure S1:** Flowchart of the study population including selection and exclusion criteria

# **Supplementary Methods S1:** Description of RII and SII measurement construction

The Relative Index of Inequality (RII) was estimated using a negative binomial regression model where the income rank probability served as the main predictor variable. Each individual’s income rank probability is computed as:

$$R\text{i,s,a,y}=\frac{rank\text{i,s,a,y}-1}{N\text{s,a,y }-1}$$

Where:

- *R_i,s,a,y_=* Income rank probability for individual *i* in sex (*s*), age (*a*), and year (*y*) subgroup.
- *Rank_i,s,a,y_=* Rank of individual *i* within the income distribution of their subgroup, where 1 represents the highest income.
- *N_s,a,y_=* Total number of individuals in the subgroup.

Subtracting 1 from the both numerator and denominator ensures that the most advantaged individual (highest income) has an income rank probability of 0 and the most disadvantaged individual (lowest income) has an income rank probability of 1.

The Slope Index of Inequality (SII) represents the absolute difference in mortality across the full income distribution, comparing the estimated lowest and highest income positions while accounting for absolute mortality rates. The SII is derived from the RII and age-standardised mortality rates (ASMR) and is calculated using the formula:

$$SII= 2\times ASMR \times\frac{RII-1}{RII+1}$$

To compute the population attributable fraction (PAF), we applied the formula shown below. We first subtracted the predicted probability of all-cause mortality in the absence of external causes, *P(Y^X^ = 1),* from the overall predicted probability of all-cause mortality, *P(Y = 1)*. The resulting difference was then divided by the overall predicted probability of all-cause mortality.

$$PAF=\frac{P\left( Y=1 \right)-P\left( Y^{X}=1 \right)}{P\left( Y=1 \right)}$$

This calculation was performed separately for each external cause category.


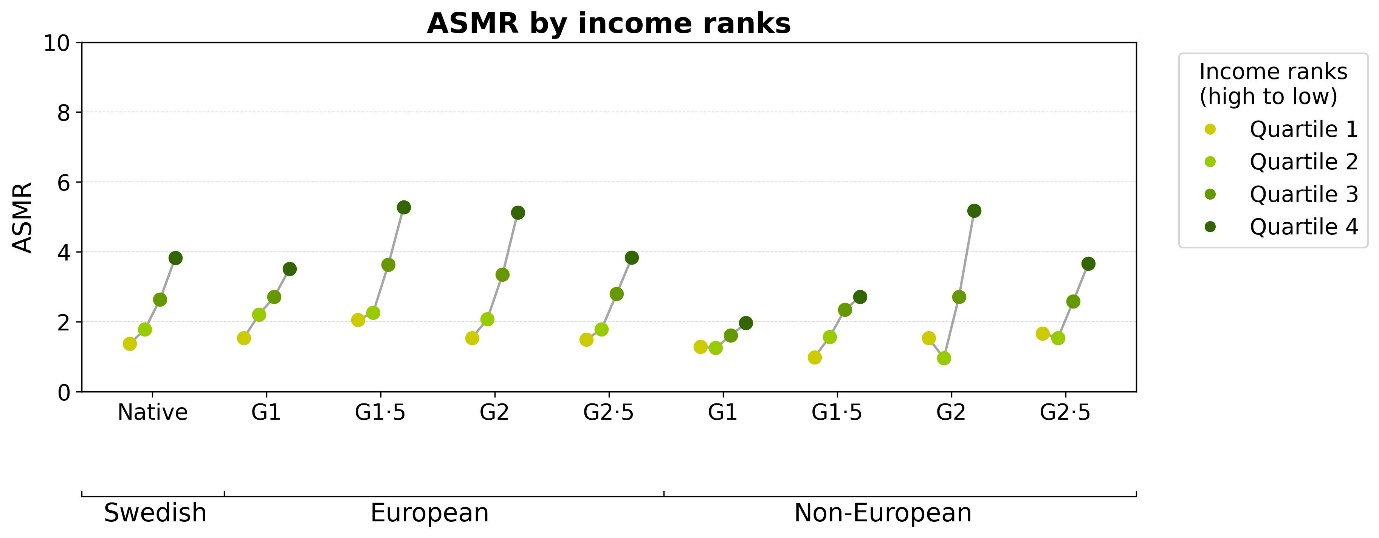


# **Supplementary Figure S2:** Age-standardised mortality rates per 1 000 person-years (ASMR) by income rank quartiles (men, 25-64 years).


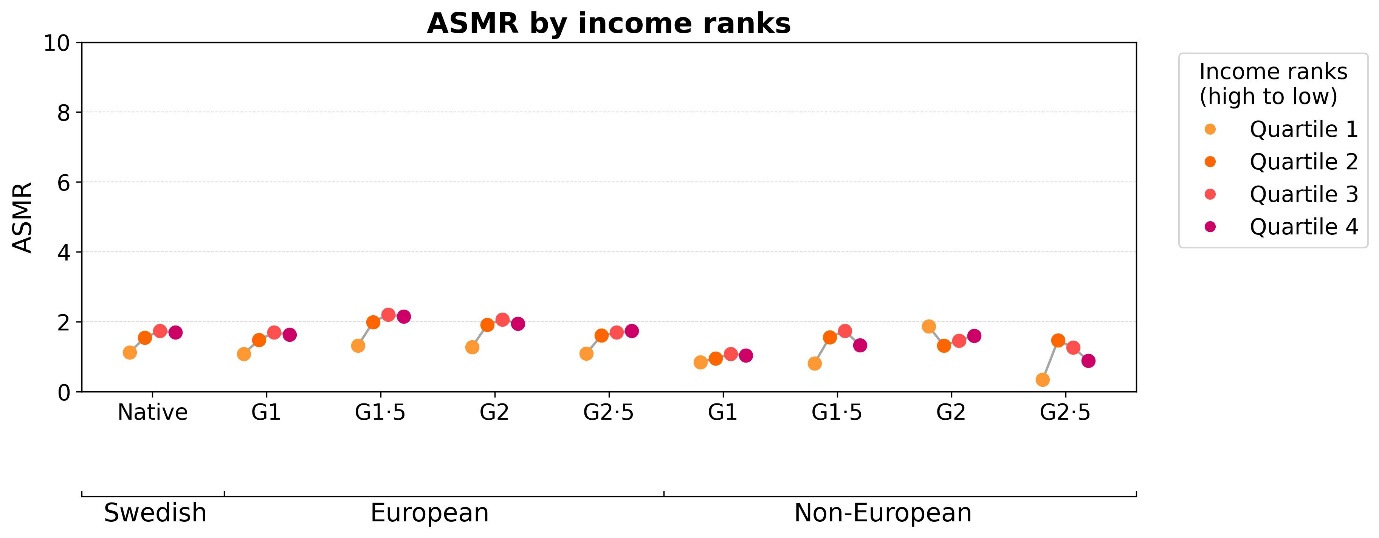


# **Supplementary Figure S3:** Age-standardised mortality rates per 1 000 person-years (ASMR) by income rank quartiles (women, 25-64 years).

# **Supplementary Table S2:** Slope Indices of Inequality (SII; per 1 000 person-years) and Relative Indices of Inequality (RII) in mortality obtained from negative binomial regressions; men 2004 to 2018

|  | **Men - unweighted** | | | | |  | **Men – weighted ^(a)^** | | | | |
| --- | --- | --- | --- | --- | --- | --- | --- | --- | --- | --- | --- |
|  | **SII** | **95% CI** |  | **RII** | **95% CI** |  | **SII** | **95% CI** |  | **RII** | **95% CI** |
|  |  |  |  |  |  |  |  |  |  |  |  |
| *Native-born majority* | 2·95 | 2·82; 3·08 |  | 4·73 | 4·36; 5·12 |  | 2·95 | 2·82; 3·08 |  | 4·73 | 4·36; 5·12 |
| *European* |  |  |  |  |  |  |  |  |  |  |  |
| G1 | 2·24 | 1·63; 2·83 |  | 2·39 | 1·88; 3·04 |  | 2·42 | 1·8; 3·01 |  | 2·59 | 2·02; 3·32 |
| G1·5 | 4·17 | 3·4; 4·89 |  | 4·33 | 3·26; 5·76 |  | 4·21 | 3·44; 4·92 |  | 4·41 | 3·31; 5·87 |
| G2 | 4·31 | 3·75; 4·82 |  | 6·39 | 4·87; 8·38 |  | 4·3 | 3·7; 4·84 |  | 6·32 | 4·71; 8·47 |
| G2·5 | 3·07 | 2·6; 3·51 |  | 4·69 | 3·64; 6·04 |  | 2·93 | 2·44; 3·39 |  | 4·25 | 3·29; 5·49 |
| *Non-European* |  |  |  |  |  |  |  |  |  |  |  |
| G1 | 0·89 | 0·41; 1·38 |  | 1·7 | 1·28; 2·26 |  | 1·31 | 0·82; 1·78 |  | 2·22 | 1·65; 2·99 |
| G1·5 | 3·26 | 2·31; 4·19 |  | 7·65 | 4·73; 12·39 |  | 2·35 | 0·59; 3·91 |  | 3·49 | 1·4; 8·71 |
| G2 | 4·35 | 2·41; 6·28 |  | 11·47 | 5·18; 25·39 |  | 4·14 | 0·62; 6·56 |  | 8·99 | 1·42; 57·08 |
| G2·5 | 3·39 | 1·94; 4·79 |  | 6·72 | 3·4; 13·26 |  | 2·35 | 0·04; 4·51 |  | 3·11 | 1·02; 9·49 |

1. Weighted by age-distribution of the native-born majority

# **Supplementary Table S3:** Slope Indices of Inequality (SII; per 1 000 person-years) and Relative Indices of Inequality (RII) in mortality obtained from negative binomial regressions; women 2004 to 2018

|  | **Women - unweighted** | | | | |  | **Women – weighted ^(a)^** | | | | |
| --- | --- | --- | --- | --- | --- | --- | --- | --- | --- | --- | --- |
|  | **SII** | **95% CI** |  | **RII** | **95% CI** |  | **SII** | **95% CI** |  | **RII** | **95% CI** |
|  |  |  |  |  |  |  |  |  |  |  |  |
| *Native-born majority* | 0·97 | 0·85; 1·09 |  | 1·96 | 1·8; 2·12 |  | 0·97 | 0·85; 1·09 |  | 1·96 | 1·8; 2·12 |
| *European* |  |  |  |  |  |  |  |  |  |  |  |
| G1 | 0·47 | 0·04; 0·77 |  | 1·38 | 1·03; 1·67 |  | 0·47 | 0·12; 0·83 |  | 1·38 | 1·09; 1·74 |
| G1·5 | 1·25 | 0·67; 1·84 |  | 1·97 | 1·45; 2·7 |  | 1·25 | 0·67; 1·83 |  | 1·97 | 1·45; 2·68 |
| G2 | 0·94 | 0·41; 1·48 |  | 1·73 | 1·28; 2·33 |  | 1·06 | 0·53; 1·58 |  | 1·86 | 1·38; 2·5 |
| G2·5 | 0·91 | 0·49; 1·32 |  | 1·88 | 1·42; 2·48 |  | 0·98 | 0·58; 1·39 |  | 1·98 | 1·51; 2·61 |
| *Non-European* |  |  |  |  |  |  |  |  |  |  |  |
| G1 | 0·03 | -0·25; 0·32 |  | 1·03 | 0·77; 1·37 |  | 0·26 | -0·05; 0·59 |  | 1·3 | 0·95; 1·79 |
| G1·5 | 0·08 | -0·48; 0·94 |  | 1·06 | 0·63; 1·8 |  | 0·66 | -0·39; 1·94 |  | 1·64 | 0·69; 3·86 |
| G2 | -0·02 | -0·84; 2·15 |  | 0·99 | 0·36; 2·74 |  | -0·78 | -1·49; 2·78 |  | 0·61 | 0·09; 4·0 |
| G2·5 | 0·75 | -0·03; 1·71 |  | 2·38 | 0·95; 5·94 |  | 0·68 | -0·15; 1·72 |  | 2·20 | 0·79; 6·11 |

1. Weighted by age-distribution of the native-born majority


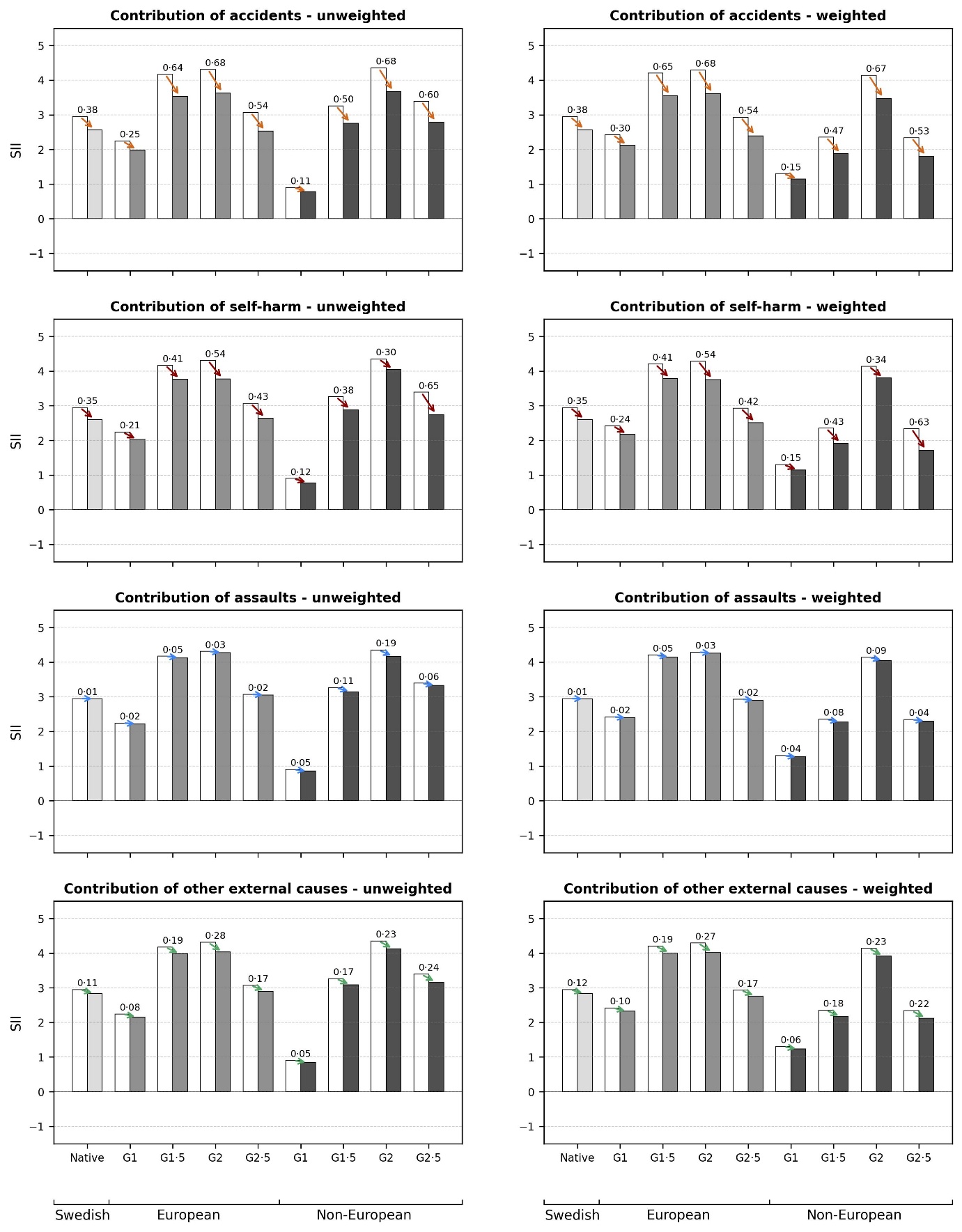


# **Supplementary Figure S4:** The contribution of specific external causes of death in Slope Indices of Inequality (SII), men aged 25-64. The values displayed above the bars show the absolute changes in the SII (expressed as mortality rates per 1 000 person-years) before and after accounting for the contribution of specific external causes of death within each nativity group.


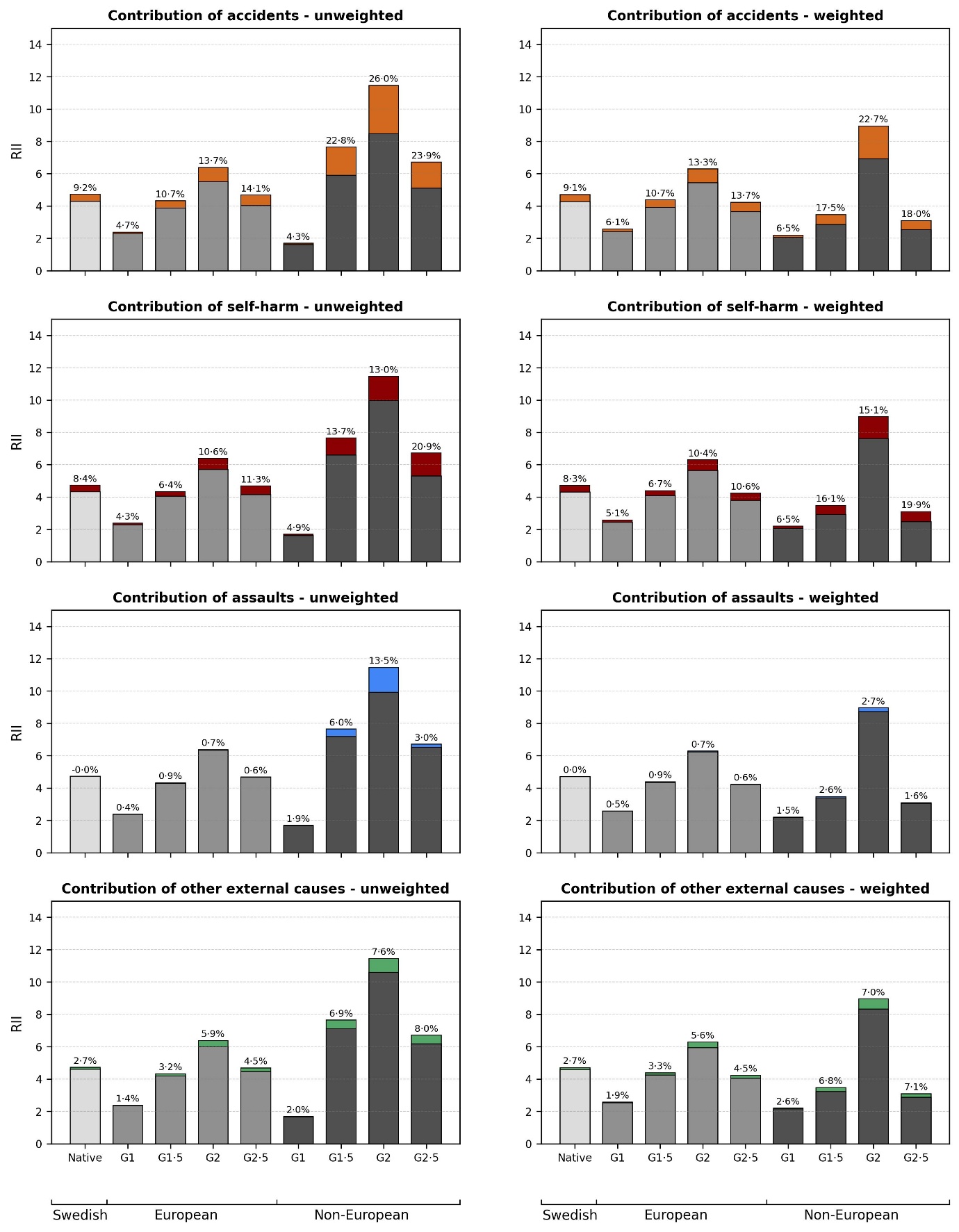


# **Supplementary Figure S5:** The contribution of specific external causes of death in Relative Indices of Inequality (RII), men aged 25-64. The percentage of the RII attributable to specific external causes of death is displayed above the bars for each nativity subgroup.


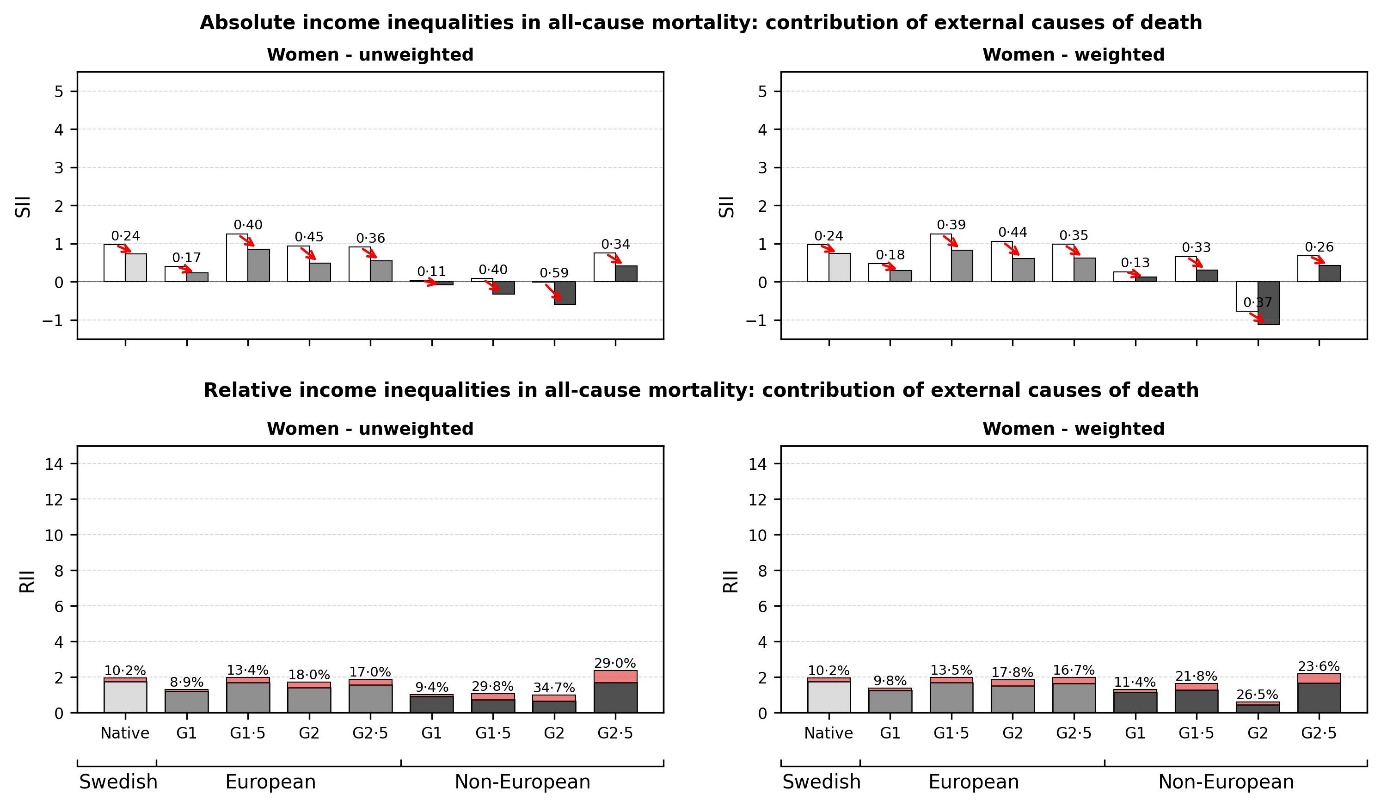


# **Supplementary Figure S6:** The contribution of external causes in Slope Indices of Inequality (SII) and Relative Indices of Inequality (RII) in all-cause mortality by nativity background in women, aged 25-64. The absolute portion of the SII and percentage of the RII attributable to external causes of death is displayed above the bars for each nativity subgroup.


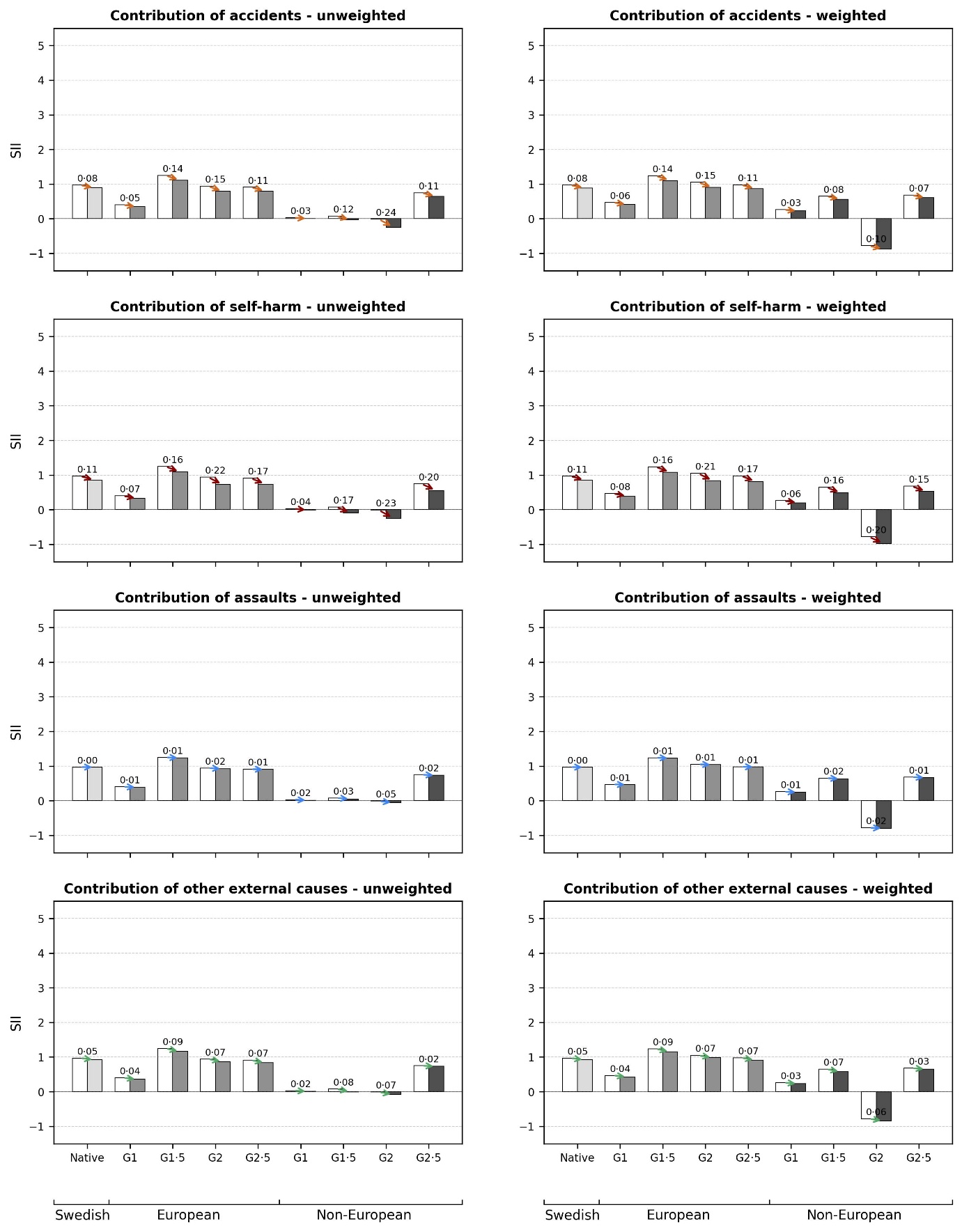


# **Supplementary Figure S7:** The contribution of specific external causes of death in Slope Indices of Inequality (SII), women aged 25-64. The values displayed above the bars show the absolute changes in the SII (expressed as mortality rates per 1 000 person-years) before and after accounting for the contribution of specific external causes of death within each nativity group.


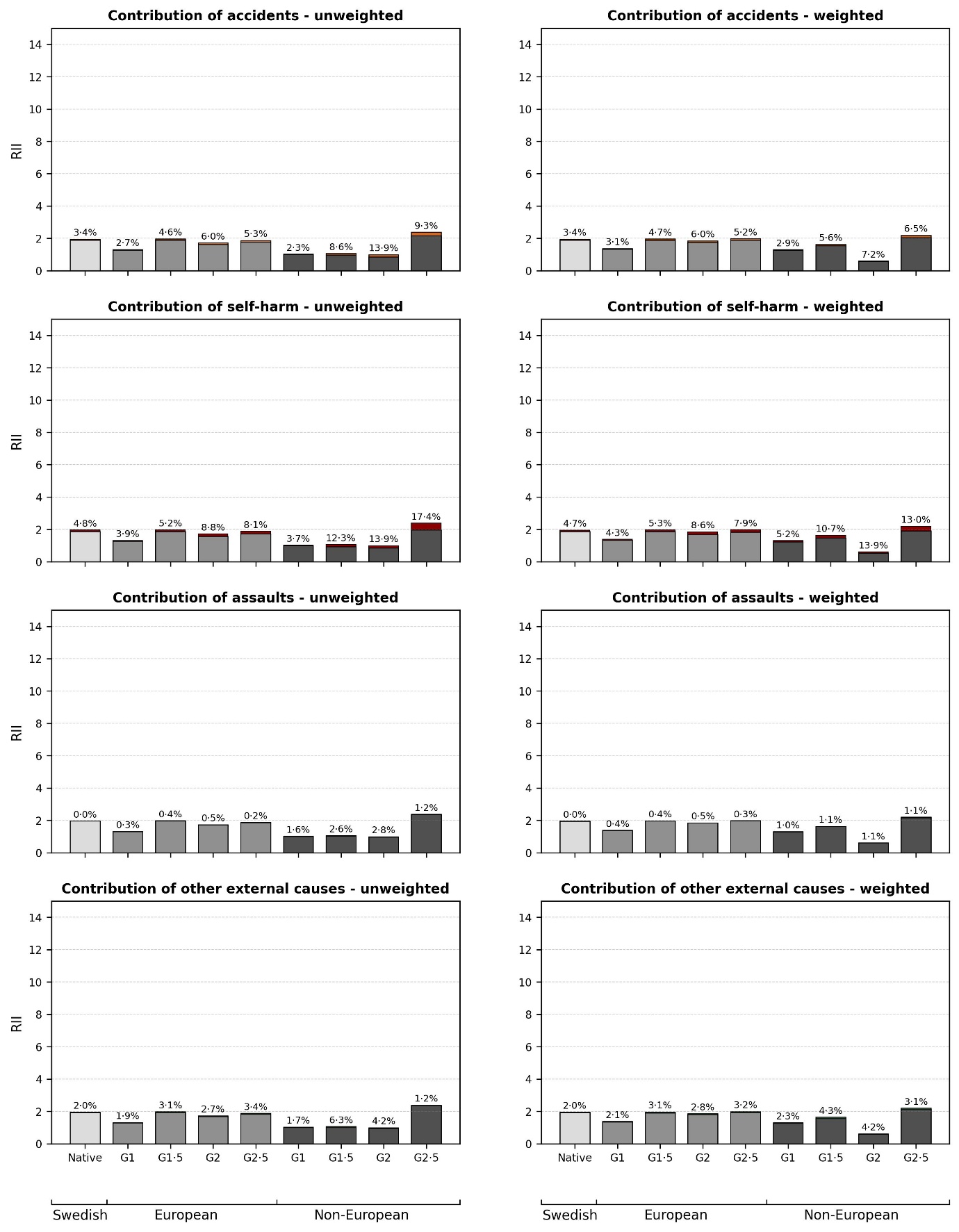


# **Supplementary Figure S8:** The contribution of specific external causes of death to Relative Indices of Inequality (RII), women aged 25-64. The percentage of the RII attributable to specific external causes of death is displayed above the bars for each nativity subgroup.

# **Supplementary Table S4:** Slope Indices of Inequality (SII; per 1 000 person-years) and Relative Indices of Inequality (RII) in mortality obtained from negative binomial regressions; men and women 2004 to 2018; adjusted for education

|  | **Men** | | | | |  | **Women** | | | | |
| --- | --- | --- | --- | --- | --- | --- | --- | --- | --- | --- | --- |
|  | **SII** | **95% CI** |  | **RII** | **95% CI** |  | **SII** | **95% CI** |  | **RII** | **95% CI** |
|  |  |  |  |  |  |  |  |  |  |  |  |
| *Native-born majority* | 2·65 | 2·52; 2·78 |  | 3·81 | 3·54; 4·11 |  | 0·64 | 0·53; 0·75 |  | 1·54 | 1·43; 1·66 |
| *European* |  |  |  |  |  |  |  |  |  |  |  |
| G1 | 1·75 | 1·15; 2·34 |  | 1·94 | 1·55; 2·43 |  | -0·11 | -0·41; 0·24 |  | 0·93 | 0·75; 1·17 |
| G1·5 | 3·74 | 2·93; 4·49 |  | 3·54 | 2·68; 4·67 |  | 0·75 | 0·19; 1·34 |  | 1·49 | 1·11; 2·01 |
| G2 | 3·92 | 3·33; 4·48 |  | 4·95 | 3·81; 6·43 |  | 0·51 | 0·02; 1·06 |  | 1·34 | 1·01; 1·8 |
| G2·5 | 2·74 | 2·25; 3·2 |  | 3·74 | 2·93; 4·77 |  | 0·59 | 0·19; 1 |  | 1·49 | 1·14; 1·95 |
| *Non-European* |  |  |  |  |  |  |  |  |  |  |  |
| G1 | 0·5 | 0·05; 0·97 |  | 1·34 | 1·03; 1·75 |  | -0·31 | -0·54; -0·04 |  | 0·73 | 0·56; 0·96 |
| G1·5 | 2·88 | 1·89; 3·88 |  | 5·27 | 3·29; 8·46 |  | -0·48 | -0·87; 0·26 |  | 0·7 | 0·42; 1·17 |
| G2 | 4·01 | 2·01; 6·05 |  | 7·85 | 3·6; 17·12 |  | -0·68 | -1·09; 1·29 |  | 0·65 | 0·24; 1·77 |
| G2·5 | 3·05 | 1·57; 4·55 |  | 5·03 | 2·57; 9·84 |  | 0·5 | -0·21; 1·5 |  | 1·75 | 0·71; 4·35 |

# **Supplementary Methods S2:** Stata commands and Python code

************************************************************

*** Calculation of age-standardised mortality rates**

gen popsize=1

dstdize death popsize age_gr, by(sex mig_group9) using("N:\...\Standardpop2016.dta") print

************************************************************

*** Calculation of the Rank index (ridit) adjusted for year, sex, and 5-year age-bands:**

bysort year sex age_band: gen inc_N = _N if DispIncome>0 & DispIncome!=.

bysort year sex age_band inc_N: egen income_rank = rank(DispIncome), unique

gen rank_index = (income_rank-1) / (inc_N-1)

gen ridit = 1 - rank_index

************************************************************

*** Calculation of three-year average of income ranks at study entry:**

bysort IDno: gen run_var=_n if ridit!=.

gen base_years = 1 if (run_var==1 | run_var==2 | run_var==3) & ridit!=. **// marks the first three years**

bysort IDno: egen base_ridit=mean(ridit) if base_years==1

************************************************************

*** Delineation of external causes of mortality**

*** deaths without all external causes**

gen d_external = death

replace d_external=0 if death_ext_all==1

*** deaths without deaths due to accidents**

gen d_accident = death

replace d_accident=0 if death_ext_accidents==1

*** deaths without deaths due to self-harm**

gen d_selfharm = death

replace d_selfharm =0 if death_ext_selfharm==1

*** deaths without deaths due to assaults**

gen d_assault = death

replace d_assault=0 if death_ext_assault==1

*** deaths without deaths due to other external causes**

gen d_other_external = death

replace d_other_external=0 if death_external_other==1

*** Aggregation of the dataset**

collapse (lastnm) death d_all_external d_accident d_selfharm d_assault d_other_external mig_group9 base_ridit base_educ age_gr sex exp_var (sum) popsize, by(IDno) **// retains the last non-missing observations of the study variables**

************************************************************

*** Negative binomial regression model for calculation of the Relative Index of Inequality (RII)**

nbreg death c.base_ridit##i. mig_group9 i.sex i.age_gr if sex==1, exp(popsize) irr

nbreg death c.base_ridit##i. mig_group9 i.sex i.age_gr if sex==2, exp(popsize) irr

*** Incidence rate ratios represent the RII. The IRR/ RII for the reference group (native-origin Swedes) is directly derived from the coefficient of c.base_ridit. The RII for each other group is obtained by multiplying the IRR of c.ridit (reference group) by the IRR of the interaction term (c.ridit × group).**

************************************************************

*** Calculation of population attributable fractions (PAF) for external causes of death (example for men)**

*** Calculation of predicted probabilities for each submodel**

nbreg death c.base_ridit##i. mig_group9 i.sex i.age_gr if sex==1, exp(popsize) irr

predict pred_dead_m **// prediction for all causes of death**

nbreg d_all_external c.base_ridit##i. mig_group9 i.sex i.age_gr if sex==1, exp(popsize) irr

predict pred_external_m **// prediction for all causes of death without external causes**

nbreg d_accident c.base_ridit##i. mig_group9 i.sex i.age_gr if sex==1, exp(popsize) irr

predict pred_accident_m **// prediction for all causes of death without deaths due to accidents**

nbreg d_selfharm c.base_ridit##i. mig_group9 i.sex i.age_gr if sex==1, exp(popsize) irr

predict pred_selfharm_m **// prediction for all causes of death without deaths due to self-harm**

nbreg d_assault c.base_ridit##i. mig_group9 i.sex i.age_gr if sex==1, exp(popsize) irr

predict pred_assault_m **// prediction for all causes of death without deaths due to assaults**

nbreg d_other_external c.base_ridit##i. mig_group9 i.sex i.age_gr if sex==1, exp(popsize) irr

predict pred_other_external_m **// prediction for all causes of death without deaths due to other external causes**

*** Calculation of the mean for all deaths**

summarize pred_dead_m

local mean_all_deaths = r(mean)

*** Define list of external causes**

local causes accident selfharm assault other_external

*** Looping over each cause**

foreach cause of local causes {

summarize pred_`cause'_m

local mean_`cause' = r(mean)

local result_`cause' = ( `mean_all_deaths' - `mean_`cause'' ) / `mean_all_deaths'

display "PAF_men_`cause' = " `result_`cause''

}

*** Calculation of overall external cause PAF**

summarize pred_external_m

local mean_all_external = r(mean)

local result_all = ( `mean_all_deaths' - `mean_all_external' ) / `mean_all_deaths'

display "PAF_men_all_external = " `result_all'

************************************************************

*** Calculation of Slope Index of Inequality**

*** Python code:**

def calculate_sii(asmr, rii):

sii = [2 * asmr * (rii - 1) / (rii + 1) for asmr, rii in zip(asmr, rii)]

************************************************************

*** Direct age standardisation by weighting (example for men)**

clear

use "N:\...\data.dta", clear

keep if sex==1

bysort mig_group9 age_gr: gen count = _N

* Calculate the total within each nativity group

bysort mig_group9: gen total = _N

* Calculate the proportion

gen prop = count / total

save "N:\...\subsample_weight_men.dta", replace

*** Creation of age-specific proportions in native origin Swedes**

keep if mig_group9 == 1

bysort age_gr: gen count_Swe = _N

gen total_Swe = _N

gen prop_Swe = count_Swe / total_Swe

keep age_gr prop_Swe

duplicates drop

save "N:\...\proportion_men.dta", replace

use "N:\...\subsample_weight_men.dta", clear

merge m:1 age_gr using "N:\....\proportion_men.dta", keepusing(prop_Swe)

*** Generate weights for each subgroup - except for reference group (native origin Swedes)**

gen weight = .

replace weight = prop_Swe / prop if mig_group9 != 1

*** Assign weight 1 to native origin Swedes in mig_group9 variable**

gen weight_1 = weight

replace weight_1 = 1 if mig_group9==1

*** Implementation in negative binomial regression model**

nbreg death c.base_ridit##i. mig_group9 i.sex i.age_gr if sex==1 [pw=weight_1], exp(popsize) irr
